# Supplementary figures and images for: Anoikis resistance in mammary epithelial cells is mediated by semaphorin 7a
Source: Cell Death Dis. 2021 Sep 24;12(10):872. doi: 10.1038/s41419-021-04133-5 (PMC8463677; doi:10.1038/s41419-021-04133-5)

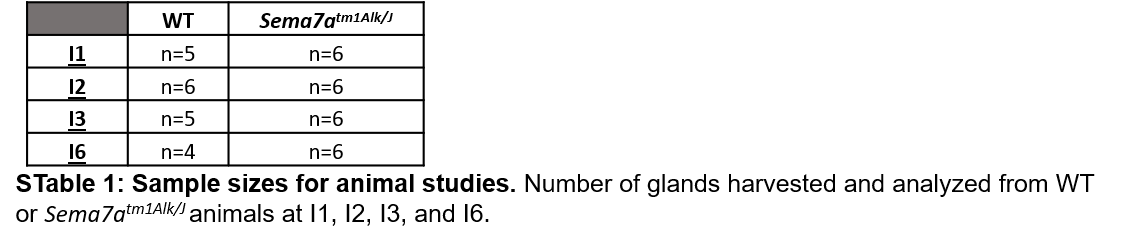

Supplement: Supplementary file 1 — STable 1: Sample sizes for animal studies. [file 41419_2021_4133_MOESM1_ESM.png]

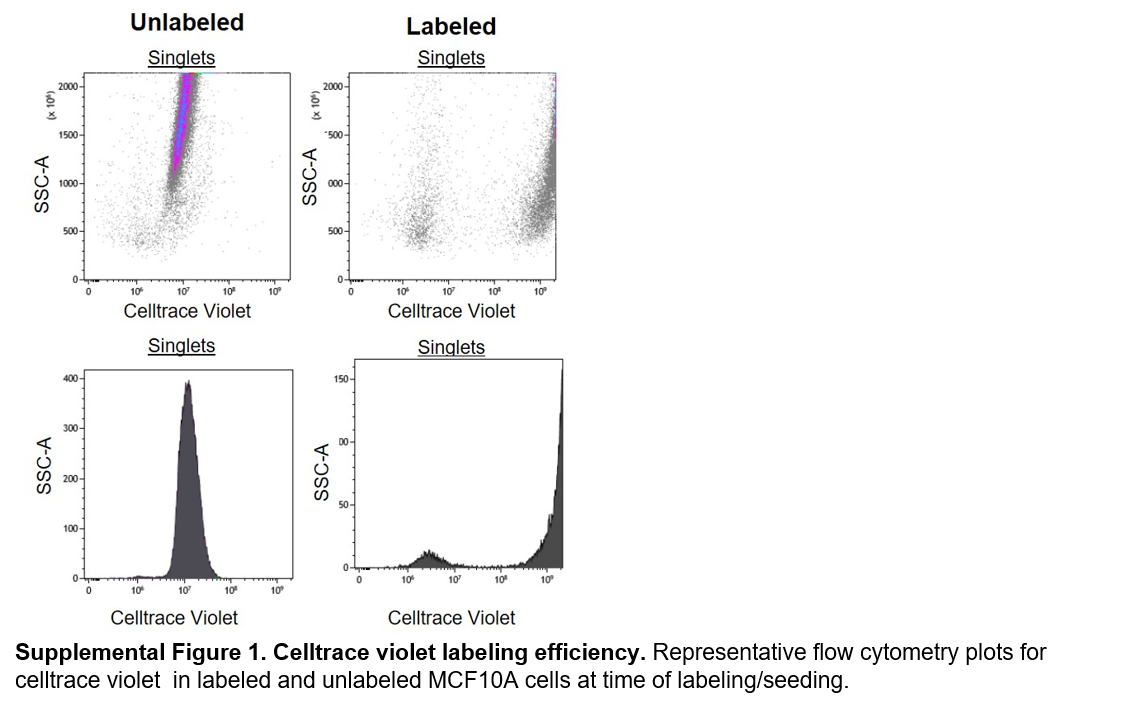

Supplement: Supplementary file 2 — Supplemental Figure 1. Celltrace violet labeling efficiency. [file 41419_2021_4133_MOESM2_ESM.png]

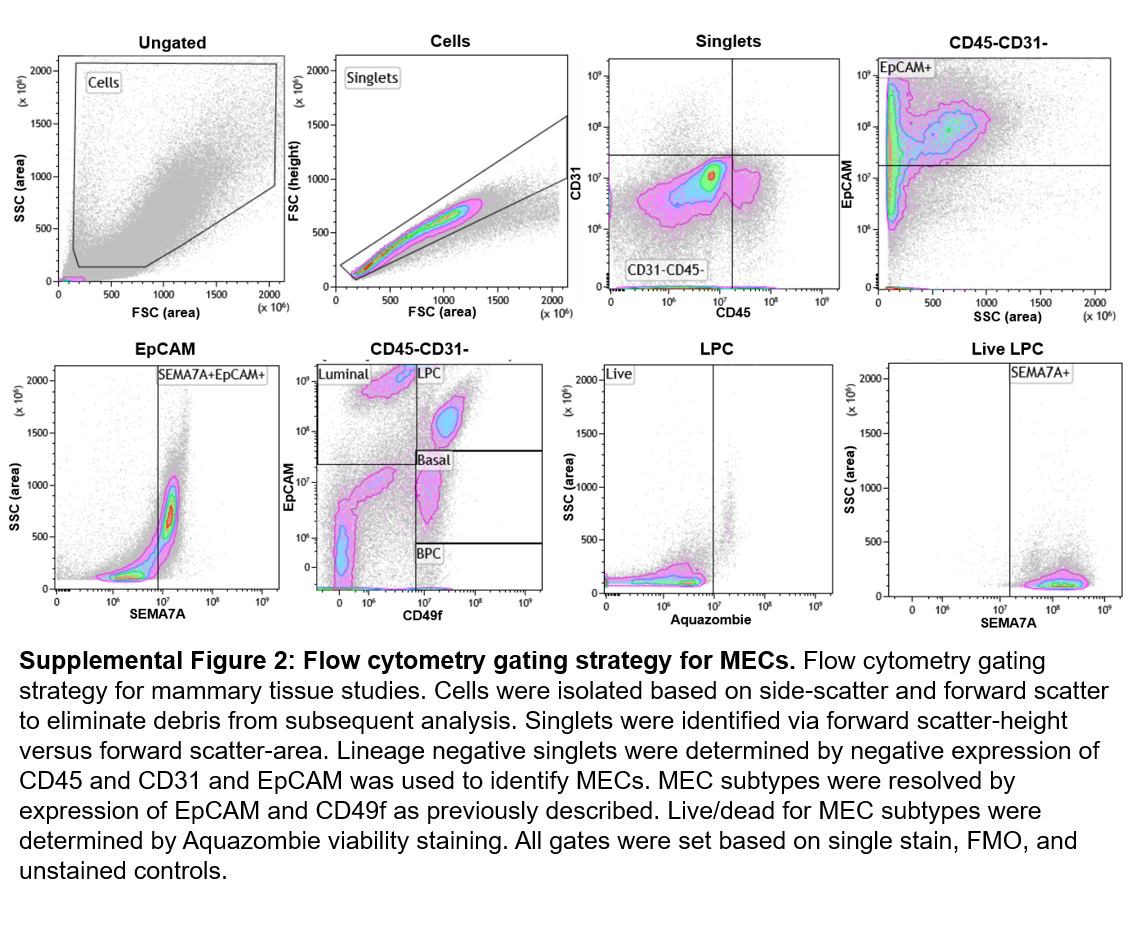

Supplement: Supplementary file 3 — Supplemental Figure 2: Flow cytometry gating strategy for MECs. [file 41419_2021_4133_MOESM3_ESM.png]

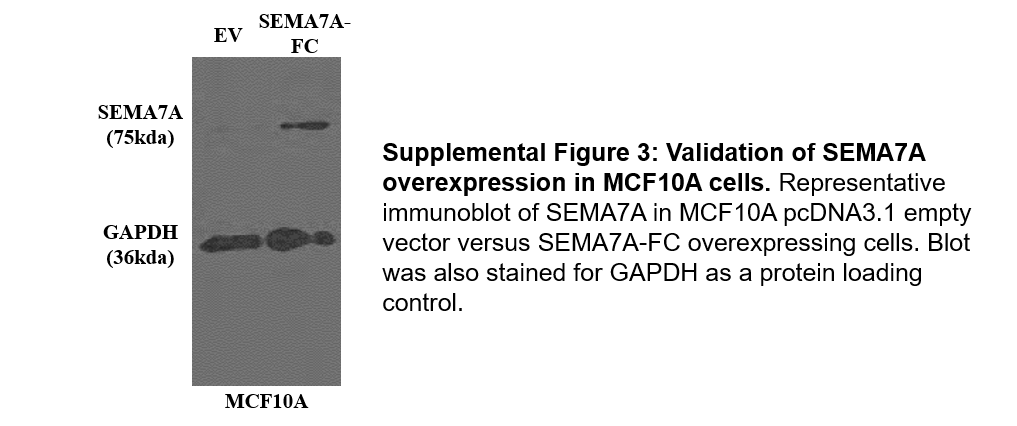

Supplement: Supplementary file 4 — Supplemental Figure 3: Validation of SEMA7A overexpression in MCF10A cells. [file 41419_2021_4133_MOESM4_ESM.png]

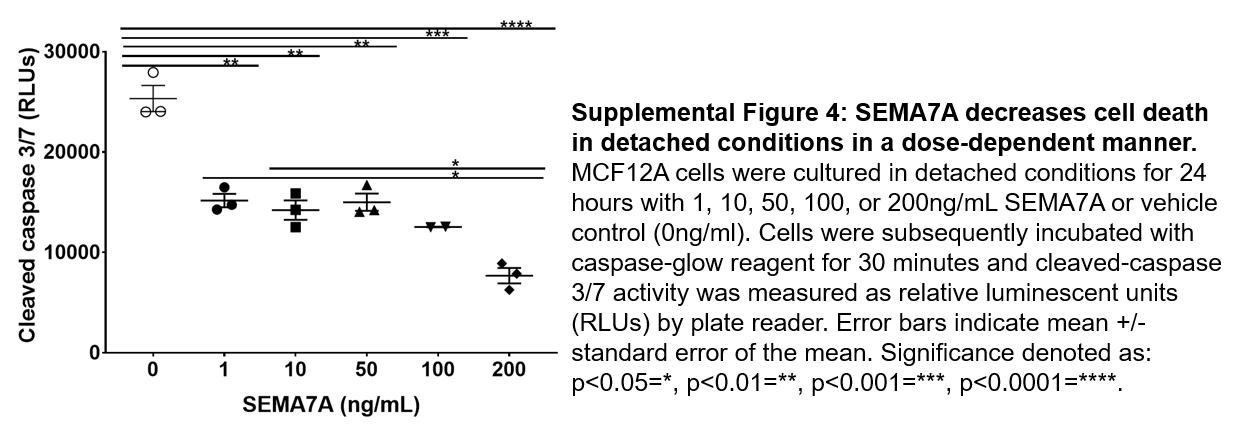

Supplement: Supplementary file 5 — Supplemental Figure 4: SEMA7A decreases cell death in detached conditions in a dose-dependent manner. [file 41419_2021_4133_MOESM5_ESM.png]

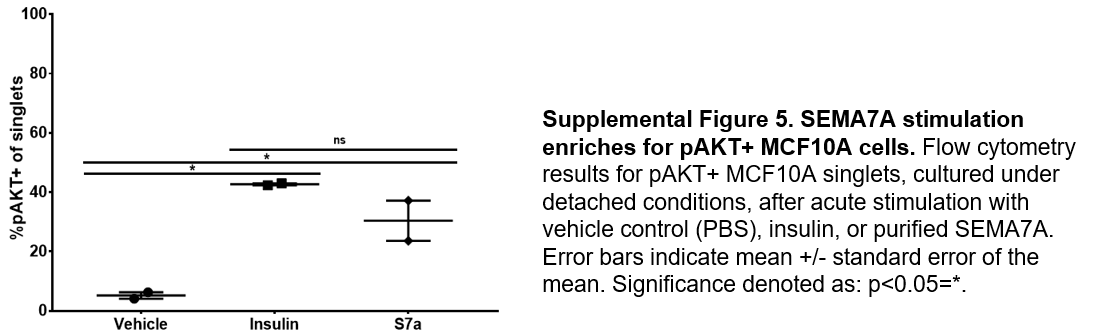

Supplement: Supplementary file 6 — Supplemental Figure 5. SEMA7A stimulation enriches for pAKT+ MCF10A cells. [file 41419_2021_4133_MOESM6_ESM.png]

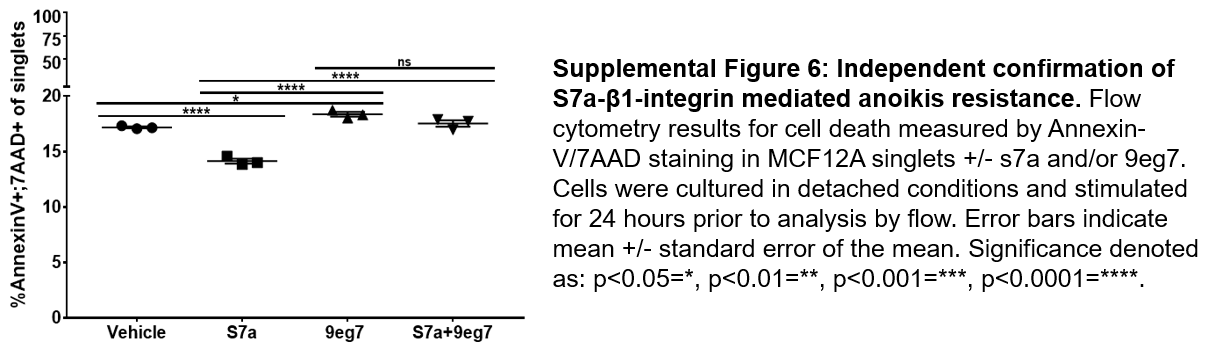

Supplement: Supplementary file 7 — Supplemental Figure 6: Independent confirmation of S7a-β1-integrin mediated anoikis resistance. [file 41419_2021_4133_MOESM7_ESM.png]

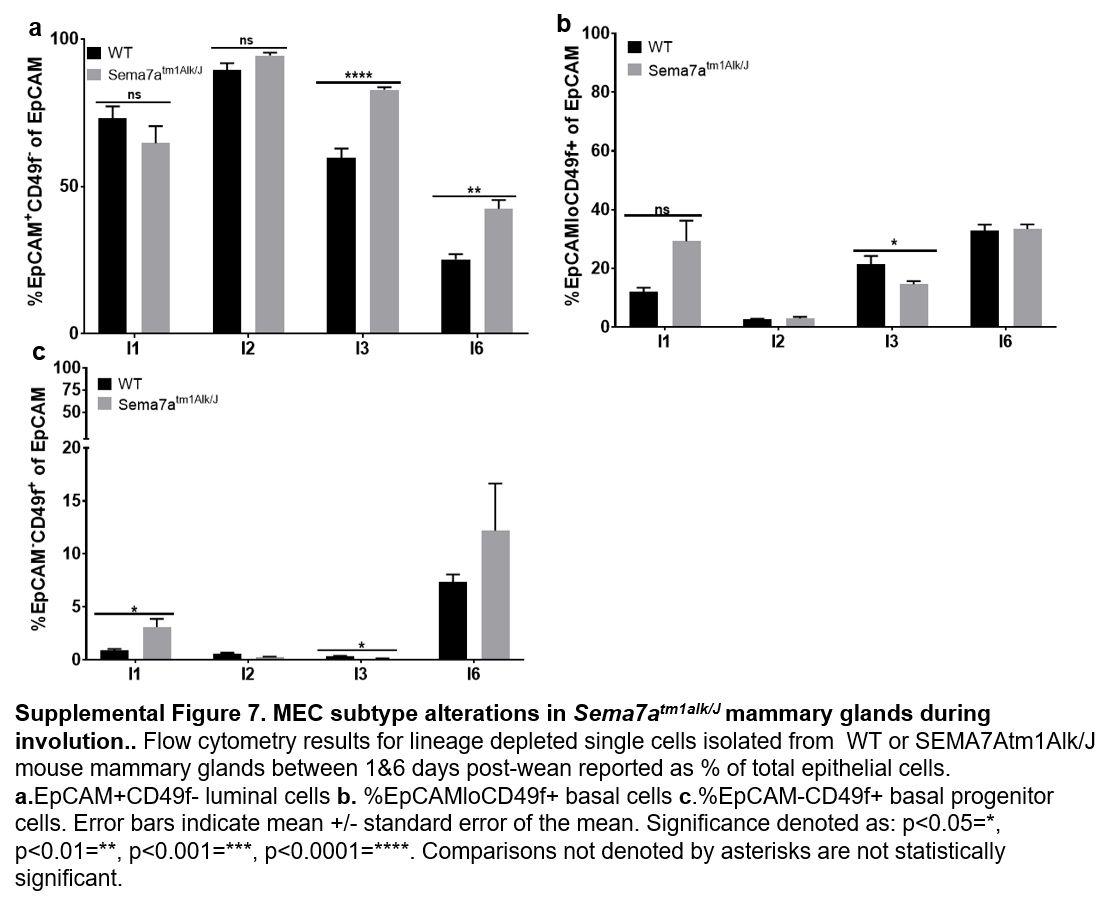

Supplement: Supplementary file 8 — Supplemental Figure 7. MEC subtype alterations in Sema7atm1alk/J mammary glands during involution. [file 41419_2021_4133_MOESM8_ESM.png]

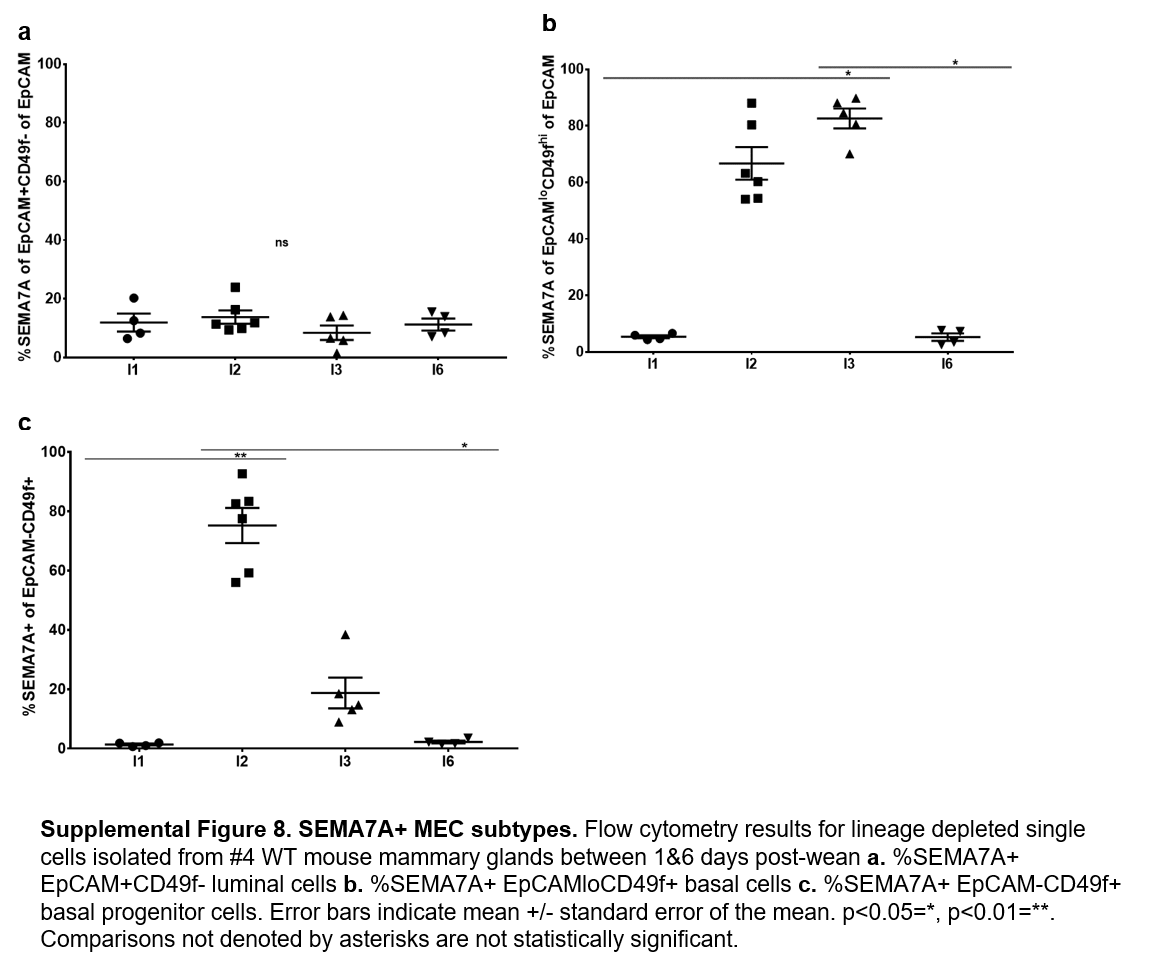

Supplement: Supplementary file 9 — Supplemental Figure 8. SEMA7A+ MEC subtypes. [file 41419_2021_4133_MOESM9_ESM.png]

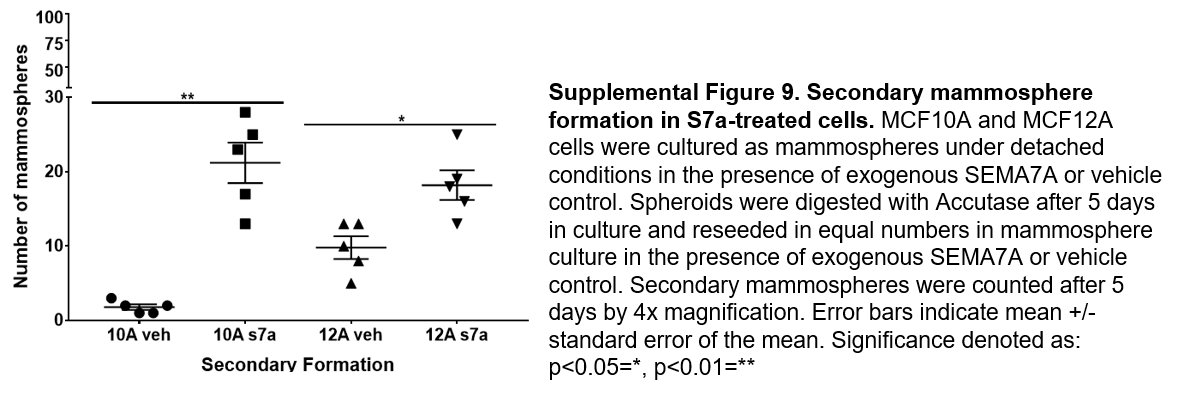

Supplement: Supplementary file 10 — Supplemental Figure 9. Secondary mammosphere formation in S7a-treated cells. [file 41419_2021_4133_MOESM10_ESM.png]

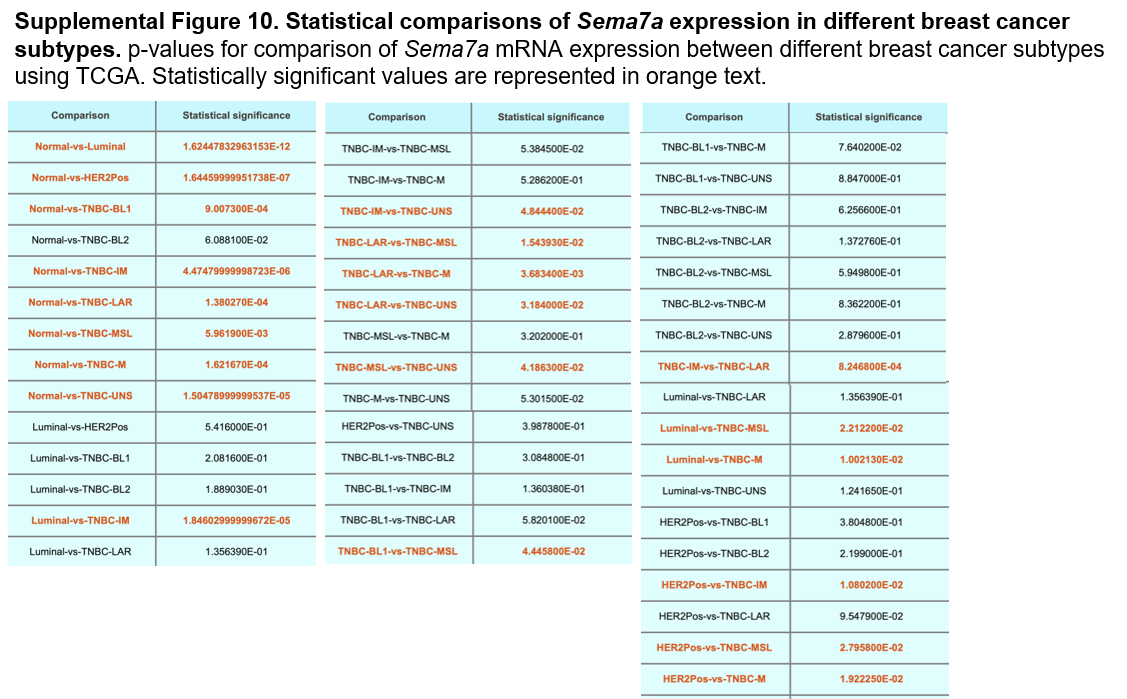

Supplement: Supplementary file 11 — Supplemental Figure 10. Statistical comparisons of Sema7a expression in different breast cancer subtypes. [file 41419_2021_4133_MOESM11_ESM.png]

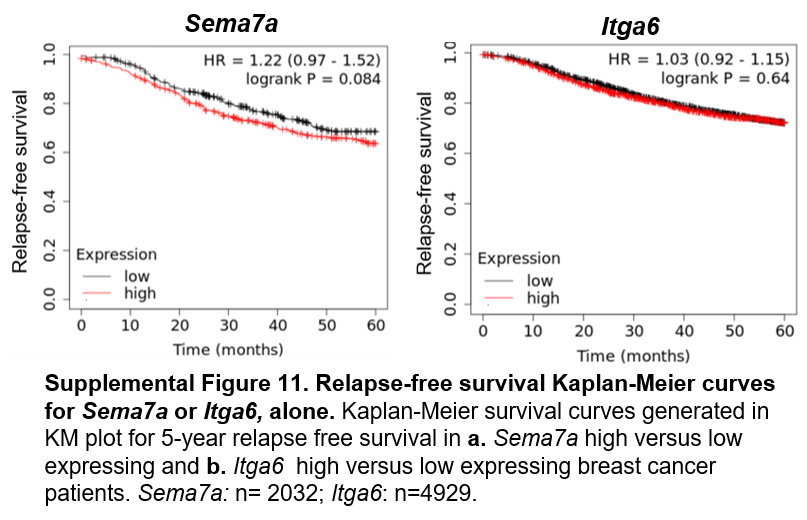

Supplement: Supplementary file 12 — Supplemental Figure 11. Relapse-free survival Kaplan-Meier curves for Sema7a or Itga6, alone. [file 41419_2021_4133_MOESM12_ESM.png]
